# Improving follow up after predictive testing in Huntington’s disease: evaluating a genetic counselling narrative group session

**DOI:** 10.1007/s12687-019-00416-9

**Published:** 2019-04-18

**Authors:** Cheryl Stopford, Mariangels Ferrer-Duch, Ramona Moldovan, Rhona MacLeod

**Affiliations:** 1 Division of Evolution and Genomic Sciences, School of Biological Science, University of Manchester, Manchester, UK; 2 Department of Clinical Genetics, Leeds Teaching Hospitals, Leeds, UK; 3 Yorkshire Regional Genetics Service, Chapel Allerton Hospital, Leeds, LS7 4SA UK; 4 Leeds University School of Medicine, University of Leeds, Leeds, UK; 5 Department of Psychology, Babeş-Bolyai University, Cluj-Napoca, Romania; 6 Manchester Centre for Genomic Medicine, St Mary’s Hospital, Manchester Academic Health Science Centre, Manchester University Hospitals NHS Foundation Trust, Manchester, UK

**Keywords:** Huntington’s, Narrative, Predictive testing, Psychosocial adjustment

## Abstract

Recently updated Huntington’s disease (HD) predictive testing guidelines emphasise clinicians’ responsibility to facilitate emotional support following testing, regardless of the result. Yet models of post-test counselling support are poorly defined. Moreover, it is unclear how these might be best delivered. In this project, a genetic counsellor and clinical psychologist developed standalone group sessions using collective narrative practices for individuals post-predictive testing. Here we present an evaluation of the experiences of one group of six people who have tested mutation positive for HD and remain pre-symptomatic. Two partners also attended the session. Observations, evaluation forms and telephone interviews were used in data collection. Interview data was available from five mutation-positive individuals and one partner. Qualitative data were analysed using a thematic framework approach. Responses were overwhelmingly positive, emphasising the importance of a specifically arranged time and space to share experiences in a structured way. This was typically the first time participants had spoken openly with someone in their situation. Narrative facilitation of discussion encouraged participants to re-discover their strengths and resiliences, with similar experiences being discovered through connections with others. The evaluation was successful in implementing group narrative interventions as part of the predictive test counselling support for Huntington’s disease. Participants suggested that the approach could be extended and adopted for other genetic conditions.

## Introduction

Huntington’s disease (HD) is an adult onset neurodegenerative disorder characterised by motor, cognitive and psychiatric symptoms. Inherited in an autosomal dominant pattern, direct mutation testing for the expanded CAG repeat that causes HD has been available since 1993 (The Huntington’s Disease Collaborative Research Group 1993). Earlier concerns had been raised around the availability of predictive testing, particularly around emotional adjustment to an adverse result. Such testing presented new challenges around personal autonomy, patient confidentiality and potential impact upon individuals and family relationships (Shaw 1987).

In response to these challenges, best practice recommendations were published (Went 1990; World Federation of Neurology Research Committee 1989), and revised in 1994 (International Huntington Association and the World Federation of Neurology Research Group on Huntington’s Chorea 1994). Whilst these guidelines are widely acknowledged as vital in the implementation of predictive testing over a relatively short time period (Harper et al. 2000; Tibben 2007), significant changes in HD research have necessitated re-evaluation (MacLeod et al. 2013). Recommendations were added pertaining to insights into HD expression (i.e. information-giving around reduced penetrance and intermediate alleles), technological advances in prenatal testing, enhanced collaborative research networks and improved understanding of psychosocial aspects of living alongside HD. Revised guidelines emphasise the importance of post-test counselling regardless of test outcome, with recognition of early coping difficulties following testing, and proactive support that enhances coping (MacLeod et al. 2013).

Twenty-five years of genetic counselling experience has highlighted the important factors that psychological support should promote. Whilst initial concerns were raised around severe adverse reactions, including prolonged depression and suicidal ideation (Benjamin et al. 1994; Kessler 1987; Kessler et al. 1984), large-scale longitudinal studies demonstrated these reactions to be uncommon (Almqvist et al. 1999; Broadstock et al. 2000; Duisterhof et al. 2001; Lawson et al. 1996; Meiser and Dunn 2000). Nonetheless, studies do reveal important effects regarding psychological adjustment and family communication. Regardless of the result, individuals demonstrate fluctuating levels of distress following testing (Codori et al. 2004; Crozier et al. 2015; Decruyenaere et al. 2003; Tibben 2007). Mutation positive carriers are believed to show the highest levels of anxiety and depression in the first 2 months following result disclosure (Almqvist et al. 2003; Bloch et al. 1992; Decruyenaere et al. 2003; Duisterhof et al. 2001; Evers-Kiebooms and Decruyenaere 1998). By contrast, peak levels of distress in individuals who receive a negative result occur after approximately 6 months (Bloch et al. 1992; Lawson et al. 1996). Systematic reviews suggest that after 1 year, rates of distress between carriers and non-carriers are comparable (Broadstock et al. 2000; Meiser and Dunn 2000).

Moreover, some studies have demonstrated enduring psychological and psychosocial adjustment effects for some patients (Decruyenaere et al. 2003; Timman et al. 2004). Such findings are complicated by the evidence that psychiatric symptoms can precede diagnosis (Julien et al. 2007; Witjes-Ané et al. 2002), yet this further underlines the importance of continuing psychological support following disclosure of a predictive test result. Significantly, one study, in which people were tested by linkage and were therefore classed as being at increased or decreased risk, highlighted that severe outcomes (e.g. suicide attempts), occurred within the first 2 years, and tended to be associated with those at decreased risk (Almqvist et al. 2003). The authors interpreted this as reflective of individuals’ difficulty integrating the new information, with a ‘crisis of identity’ in response to their new found status.

Identity adjustments are important when considering family communication. Whilst evidence suggests that most spousal relationships remain unchanged in the long-term (Codori and Brandt 1994; Decruyenaere et al. 2004), result disclosure does have the potential to damage relationships (Andersson et al. 2016; Tibben et al. 1997). Individuals may be forced to envisage a new future identity, as a dependant or carer. Alternatively the removal of long-held expectations of a mutation-positive result may be difficult to accept and cause disruption (Codori and Brandt 1994; Huggins et al. 1992). Although a mutation-negative result may remove perceived barriers and allow the prospect of new lifestyle choices, couples may need to realign previously held views (Brouwer-Dudokdewit et al. 2002; Sobel and Cowan 2000). Additionally, individuals may feel a responsibility to inform family members of their result, causing anxiety and changes to family relationships (Tibben et al. 1993; Tibben et al. 1992). The predictive testing process may therefore generate the re-evaluation of individual identity, partnership roles and family dynamics. Re-construction of these roles and relationships may be required at different points in the lifecycle, depending upon the situations that people come to face (Brouwer-Dudokdewit et al. 2002). This fluctuating impact on the individual and their wider family interactions ought to be considered within and beyond predictive testing (Sobel and Cowan 2000; Tibben et al. 1997).

Being a presymptomatic carrier of a HD mutation fosters many uncertainties. There are no assurances when symptoms will begin, or how they will manifest. This knowledge may influence decision making at various time-points, making it particularly important to consider individuals’ sense of control (Brouwer-Dudokdewit et al. 2002; Tibben 2007). Encouragement of autonomy and active support-seeking may therefore be particularly helpful in improving coping strategies (Brouwer-Dudokdewit et al. 2002; Rolland and Williams 2005). Concentration on open communication and connectedness are considered central to maintaining family relationships and reducing isolation (Decruyenaere et al. 2004; Sobel and Cowan 2000; van Oostrom et al. 2003). Work from psychotherapeutic practice and family systems therapy suggests that families who speak more openly and tell a ‘coherent story’ of their experiences allow reflection and discussion of emotions and ideas without avoidance or ‘entanglement’ in the subject (Byng-Hall 1998; DudokdeWit et al. 1998). This work underlines the genetic counsellor’s role in focusing attention on the counselee’s strengths as opposed to their weaknesses. Rather than concentrating on and pathologising ‘deficits’, benefits can be found from seeking examples of ‘resilience’ and strength within the counselee’s story (Barnard 1994).

An approach that fosters these attributes is that of narrative practice (White and Epston 1990). This approach, sometimes described as ‘re-storying’ conversations, aims to help people to separate their problems from their personal identities in a respectful, non-critical manner (Morgan 2000), and to continue developing those stories that may be neglected but potentially significant (White 2007). Through questioning and collaborative conversation, the individual is encouraged to centre themselves as the experts of their own lives. Narrative practice perceives lives as ‘multi-storied’, meaning that there are multiple ways in which experiences can be expressed and viewed. The theory respects that personal identities are actively created through interactions with others. Together, participant and the counsellor co-create a rich description of the person’s preferred account of experiences, allowing reflection upon their multiple, connected stories and their existing skills, beliefs and values, rather than concentrating on dominant, deficit-oriented narratives.

Narrative practice has been successfully employed in patient groups experiencing significant periods of distress and trauma, for example HIV-positive individuals recovering from addiction (Garte-Wolf 2011), families living with cancer and dealing with bereavement (Hedtke 2014) and people living with dementia (Young 2010). In each case, the counsellor works alongside the individual, assisting them to discover or ‘rediscover’ their existing agency, whilst acknowledging present and past difficulties. Given the ever-changing roles and identities of people living in HD families, one might anticipate that narrative practice could be an extremely valuable approach to adopt alongside genetic counselling.

In response to recommendations for improved post-test counselling support, we have developed group sessions utilising narrative practices, offered to individuals post-predictive testing. The aim was to evaluate a new clinical service improvement initiative, led by a Genetic Counsellor (RMac), and a Clinical Psychologist (MF-D), who developed standalone narrative group ‘clinics’ for people following predictive testing for HD. The study allowed for prospectively planned appraisal of the service following an initial pilot exploration with a group of individuals who had received mutation negative test results (MacLeod et al. 2017). Here we focus on a session involving individuals who had received a mutation positive test result. Keen to understand the value and feasibility of integrating narrative practices within a genetic counselling setting, we explored individuals’ experiences of a structured narrative group session.

## Methods

### Study design and context

This exploratory service evaluation focuses on qualitative outcomes. Sessions were facilitated predominantly by a Clinical Psychologist, who was not known to participants. The Genetic Counsellor, who had been involved in the predictive testing process with participants and was potentially involved in onward follow-up, also helped to facilitate the session. The evaluation was carried out independently by a student as part of an MSc Genetic Counselling project.

### Participants and recruitment

As part of a local Family Register Service, individuals are contacted every 2–3 years to prompt connection with the clinical genetics department and to provide details of advances in research or clinical care. Individuals consented to this service (*n* = 709 families) are sent review letters and research information sheets automatically. As part of this service, the initiative was initially offered to three groups, the first involving mutation negative individuals, the second being the present group and later groups involving a mix of mutation positive, negative and at-risk individuals.

Fifteen individuals who had tested mutation-positive and remained undiagnosed were purposively selected and invited to attend a 2-h group session in June 2016. The Genetic Counsellor invited people who were known to the family register service and following recent contact with the genetics department (e.g. at the time of their annual medical review). Reasons for declining to participate included dislike of group activities, difficulty taking time off work (in one case starting Pre-implantation Genetic Diagnosis treatment) and lack of availability due to holiday. Two participants expressed strong wishes to bring their partners who had been through the counselling process with them. Following this request all participants were told they could bring someone with them if they wished. The final group comprised eight people: six mutation-positive individuals (three males, three females) and two male partners (not at risk of HD).

### Structured narrative exercise

The group session utilised an adaptation of the ‘Tree of Life’, an approach which uses parts of a tree as metaphors representing the different aspects of peoples’ lives (Ncube-Millo and Denborough 2007). In this adaptation, the group sits in a circle, with the facilitator next to a picture of a bare tree painted onto a board. Facilitators do not utilise ‘scripts’ but the session takes a clear guided structure, which is described in full in an earlier paper (Macleod et al. 2017). Narrative theory guides the facilitator to use questions that help participants to broaden their perspectives on how they see themselves and a range of possibilities for living their life.

Within the session, the facilitator encourages participants to think about HD in the context of a tree metaphor. As trees may have to face hazards, such as storm, or disease, HD could be named as one of the ‘big storms’ of life.

**Fig. 1:**
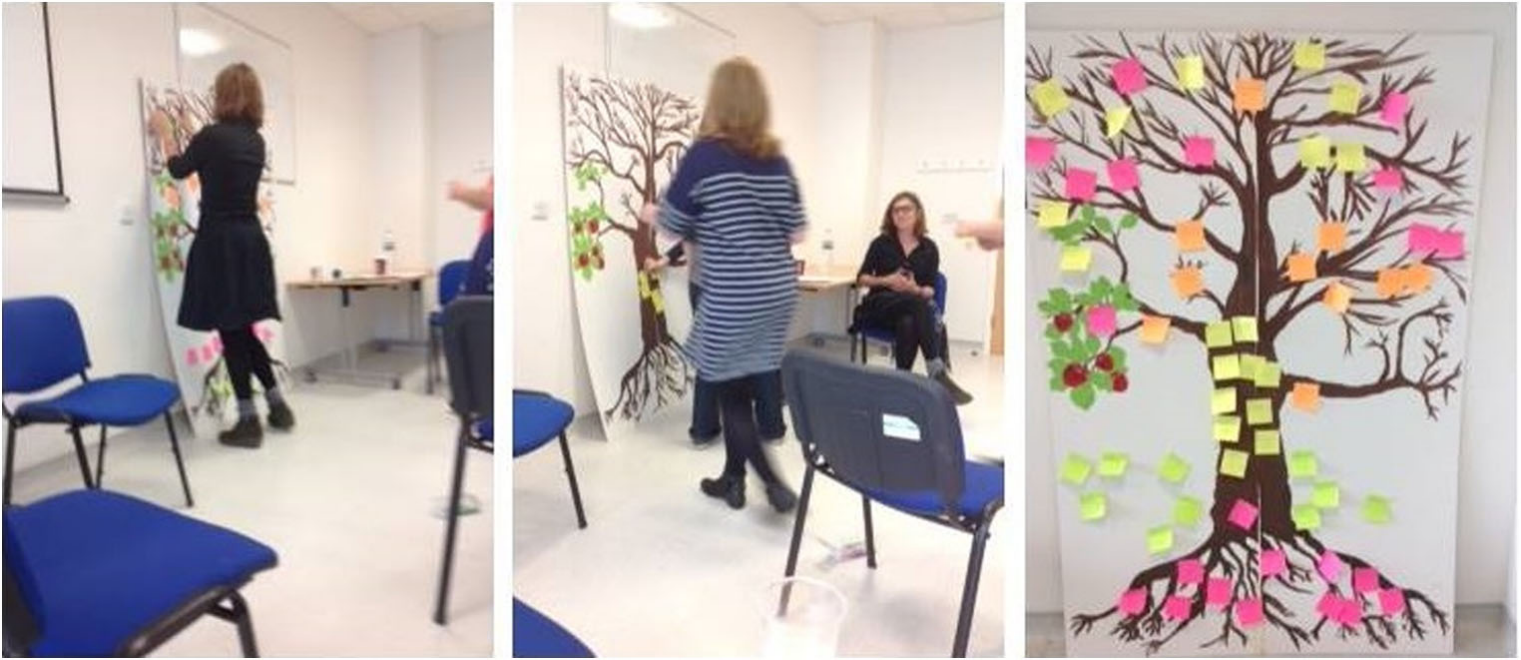
The Tree of Life in action. There are three photographs, each showing the room where the Tree of Life exercise took place. There are some chairs, arranged to create a semi-circle. A tree, with bare roots and branches, is painted on a large board, which leans against the wall. In the first photo, the facilitator is placing a post-it note onto the branches of the tree. The second photo shows the other facilitator walking towards the tree in order to place a post-it note onto the trunk. The third photo shows the tree at the end of the session, when it has been covered in post-it notes which represent the contributions of the group as they reflect on their background, daily lives, skills, hopes, important figures and contributions that others have made

Participants are asked to provide examples of different facets of their lives in relation to the tree’s parts, each time asking them to write their responses on coloured post-it notes, and attach them to the corresponding part of the ‘tree’ (Fig. 1).

Parts of the tree are considered in turn:

- The roots: representing heritage and background (e.g. family, friends, places)
- The ground: symbolising important aspects of daily life and everyday routines (e.g. running, music, work)
- The trunk: representing participants’ skills and what helps them to get through difficult times (e.g. being active, ‘being there’ for people)
- The branches: representing hopes, wishes, and dreams (e.g. enjoying life, having fun, treatments for HD)
- The leaves: symbolising individuals who are special, important and influential (including those who have passed away, famous idols, pets or even fictional characters)
- The fruits: representing the ‘gifts’ or contributions that others have made to the participants’ lives (e.g. time, positivity)

After written responses are stuck onto the tree for each part, the facilitator invites discussion around the descriptions that participants find important and sustaining. For instance, someone writes “humour”. The facilitator asks “where did you learn the importance of humour?”, “why is humour important to you? and “who else do you know that might have used humour?”

Once the tree is completed it offers a different ‘place’ for participants to stand, allowing them to view their reflections from a different perspective. They are asked to consider naming the tree, reflecting upon what it looked like at the beginning and the discussions that they have had during the session. Finally, participants are asked to reflect on the session in general. A document is created and distributed to participants, to remind them of the themes co-created during the exercise.

### Data collection and outcome measures

Participants were asked to complete written feedback forms at the end of the session. Both open (e.g. ‘What are your thoughts on today’s session?’) and closed (e.g. ‘Did you feel that the session was useful?’) questions were asked. Free-text comments were encouraged to explore participants’ experiences of taking part. They were also invited to take part in telephone interviews after a 6–8-week period. Interviews were semi-structured. The topic guide covered description of the session experience, reaction to the session and general psychological well-being and mood. Questions were asked such as the following:

- Can you tell me a little bit about how things have been for you since we met (at the session)?
- Have there been any times that you felt that you could draw from things that were discussed?
- If so, what kind of things did you find helpful to draw from?
- What were the most helpful things to take away?
- Were there things in the session that you felt were unhelpful?

Participants provided verbal consent to audio recording.

Observations were recorded during the session using detailed field-notes. This helped to provide richer context for data interpretation; however, the present study focuses on the interview and evaluation form data.

Participants also completed Generalised Anxiety Disorder-7 (GAD7) and Patient Health Questionnaire-9 (PHQ9) scales pre-session and 2 weeks later. These standardised tools were chosen as they are widely used in primary care settings as reliable, valid and brief methods of assessing mood and anxiety states, with high levels of sensitivity and specificity (Kroenke et al. 2010).

### Data analysis

**Fig. 2:**
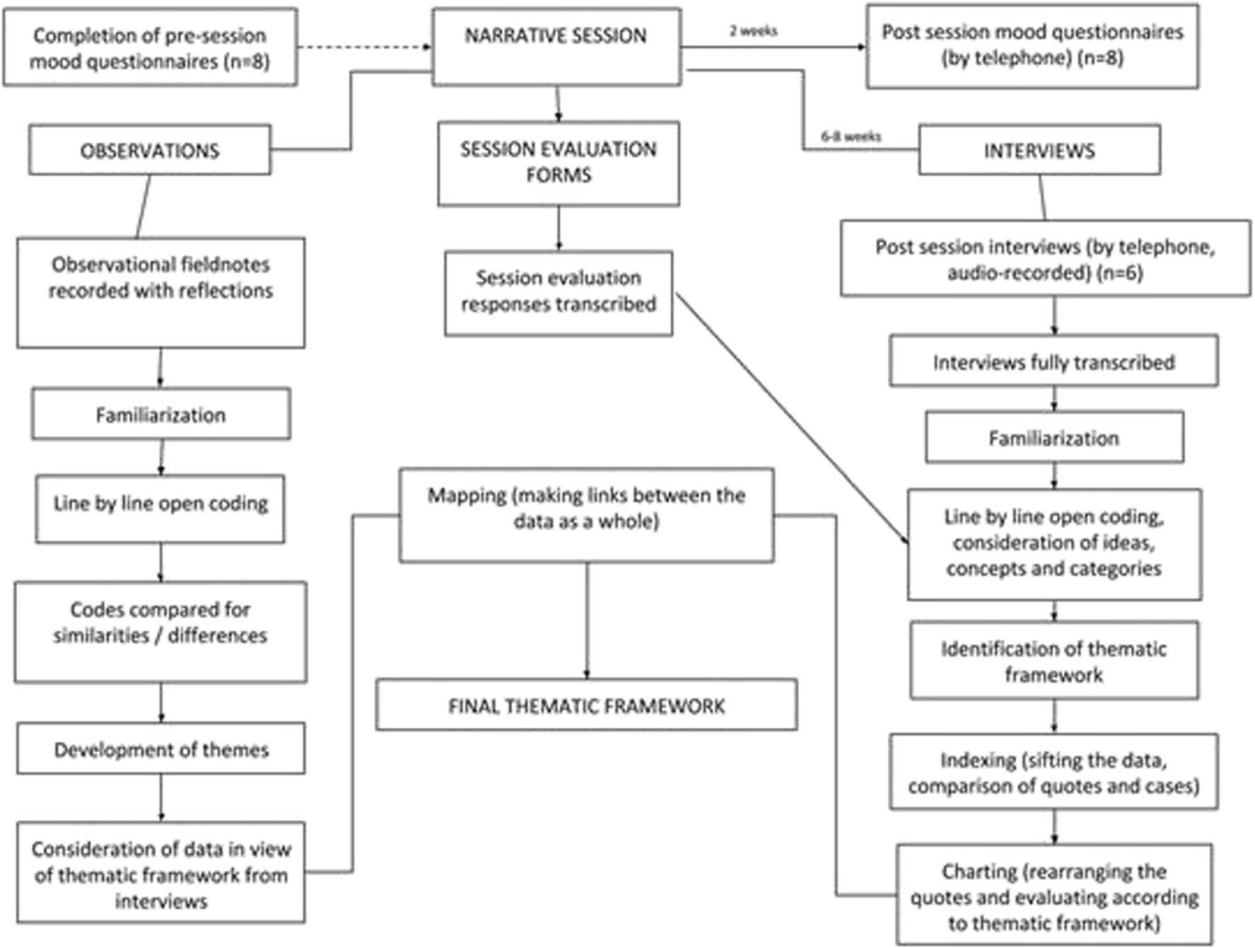
A process map of data collection and qualitative analyses. The process map illustrates the route of the qualitative analysis. Central to the diagram is the narrative session and analysis of the evaluation forms. Transcribed responses were analysed alongside the interview transcripts. The diagram shows how the interview and observation data were considered in parallel, with development of themes from the observations helping to inform the final thematic framework

Written comments from the feedback forms were transcribed for analysis. Recorded interviews were transcribed, with detailed reflections documented during data collection to assist interpretation. A process diagram is presented in Fig. 2.

Qualitative data was managed using NVivo software to ensure a clear audit trail from raw data to theme extraction. Framework analysis (Ritchie and Spencer 1994) was employed to extract and compare themes from the feedback forms and telephone interviews. This method takes a guided approach from initial data management to the development of descriptive and explanatory accounts. It allows cross-examination at both case and theme level for greater transparency.

Following initial familiarisation, interview transcripts were coded at a line-by-line level. Initial primary codes were listed and commonalities were examined to determine a coding ‘framework’. This thematic framework was then applied to individual transcripts, with coding matrices being produced using NVivo software to index and chart the data, comparing, describing and examining individual participant quotes within each theme. Throughout the analysis the interviewer (CS) and Genetic Counsellor (RM) reflected upon the transcripts independently and met to discuss and refine potential themes. The final framework was developed after discussion of the interviewer’s independently produced themes.

Alongside interview data, observational fieldnotes were examined using a thematic approach. The fieldnotes, rich in content regarding the process and structure of the session, were described using inductive, data-driven, manifest codes, which were then refined and compared in order to add to the development of the themes.

In view of the small sample size, descriptive statistics are used to illustrate the pre- and post-session scores from the quantitative scales.

## Results

### Demographics and mood scale scores

Participant characteristics are presented in Table 1. All attendees completed session evaluation forms and mood questionnaires and agreed to take part in interviews. Five mutation positive individuals and one partner were interviewed 6 weeks after the narrative group. The remaining two individuals were unavailable by telephone (one had changed his telephone number and another was busy at work).

**Table 1:** Participants’ demographic information

Participant	Genetic status	Gender	Age	Marital status	Interviewed?
1	Mutation present	Female	34	Married (to P6)	Y
2	Mutation present	Female	30	Partnership	Y
3	Mutation present	Female	19	Partnership (with P8)	Y
4	Mutation present	Male	32	Single	Y
5	Mutation present	Male	45	Married	Y
6	Not tested (partner)	Male	34	Married (to P1)	Y
7	Mutation present	Male	29	Partnership	N
8	Not tested (partner)	Male	25	Partnership (to P3)	N

Levels of anxiety and depression, as scored by standardised mood questionnaires, were low both before and after the session, with participants typically scoring within the ‘mild’ range. As Table 2 illustrates, there were no apparent positive or negative changes following the session. Given the small number of participants, and potential for floor effects (five participants scored 0 or 1 pre- and post-session), statistical tests were not employed.

**Table 2:** Participants’ median and range scores on standardised mood questionnaires, prior to and following the narrative session

	GAD-7 (anxiety)		PHQ-9 (mood/depression)	
	Pre-session	Post-session	Pre-session	Post-session
Median	0.5	0	1	0
Range	0–5	0–5	0–6	0–6

### Participant experience

Analysis of interviews and evaluation forms revealed three main themes: experiences during the session, post-session reflections and thoughts for the future.

### Theme 1—experiences during the session

Participants described how they had felt within the group setting. They expressed the importance of structure in facilitating communication and highlighted differences between organised and unstructured contact. They named the activities within the session, remarking upon their value. Four subthemes were identified.

#### **‘**A safe and welcoming space’

Participants described the atmosphere of the session positively, emphasising feelings of safety and comfort.

“it were welcoming and people were friendly, and it was …, helpful”. (P1)

This atmosphere aided them to engage and speak freely with their newly acquired acquaintances, even on difficult subjects such as bereavement, illness and challenging family relationships.

“I didn’t feel awkward in there at all—I felt quite comfortable, because you didn’t have to say anything that you didn’t want to say, it was all stuff that you wanted to say” (P2)

“I think, to do it in a small group like that, you feel quite open to speak out about it, [ … ] when you know there’s other people in the same position as you.” (P3)

#### ‘Group dynamics’

Participants compared themselves with fellow group members, highlighting differences in age, background and current situation. For most, the diversity amongst participants’ experiences was viewed as a way of gaining alternate perspectives.

“Everybody is different in how they see things and read things…” (P1)

“ … I thought there was a good, kind of cross section of people, who were there.” (P5*)*

“everyone’s got their own kind of diversity … I think if you’ve got a more diverse panel, as you guys had, then it does help.” (P4)

Unknown to the facilitators, two participants were acquainted with each other prior to the session. They had met on several occasions, oblivious to their shared status. Acknowledging this in the waiting room, they enlightened the facilitators at the mid-session break. Although both appeared surprised, neither reported adverse feelings towards this chance meeting. Whilst both observed that they might have felt differently had the person been someone they disliked, they remarked that the situation had in itself been helpful in demonstrating how others were living with a positive predictive test result.

“I kind of knew him but I didn’t ‘cause obviously we’ve only ever sort of bumped into each other in talks at sort of things like that, so he’s not necessarily a friend in that sense, but, if anything it just kind of re-affirms that, you know, there are other people out there you know, kind of living with the same thing.” (P5)

For one participant in particular, this seemed to be beneficial.

“… I think especially knowing about (other participant)—I think that did, yeah, kind of have an effect on me. So I think I came away from that more positive than negative, you know.” (P4)

#### ‘The importance of structure’

Whilst many participants reported that they took a relatively ‘open’ attitude regarding discussion of their genetic status, as might be expected given their choice to take part in a group activity, they were acutely aware that others in the same position might take a different view. Communication with others in the same situation had therefore been limited, and for most people, not considered at all. Participants described how this could lead to feelings of isolation.

“… I think with like, the whole Huntington’s thing, people do feel like they’re alone and there’s not a lot of people that are going through the things that they are”. (P2)

Participants highlighted the necessity of an externally organised ‘space’ and time for communication with others in the same position, noting how this would not occur naturally in day-to-day life. They spoke of the positive aspects of the session structure and how this stimulated natural conversations.

“I think you do feel quite wary about speaking to people unless it’s arranged really, in this kind of environment”. (P3)

“It was kind of well led, it was kind of nice and structured but it was kind of open enough to, sort of, talk…” (P5)

“Everything was good about it […] how they planned it out to do that tree … ” (P6)

#### ‘Encouragement of activities’

Participants described how the session had provided opportunities to engage in activities such as listening to others, and sharing stories and information about family relationships.

“I think that when I was in there, I felt like it sort of, helped me … obviously listening to other people, I think that helps you as well” (P3)

They expressed the value of comparing themselves with others in the same position. The session required them to reflect on their own and others’ ways of managing their situations. This was thought to be helpful in evaluating and considering coping mechanisms for the future.

“I think just to get people to understand how Huntington’s affects each of us, you know, in different ways … I know how it affects me, but there I would see it showcased how it affects other people.” (P4)

“Having open conversation in a frank way with strangers forces one to think and articulate internal thoughts”. (P7)

### Theme 2—post-session reflections

All attendees reported that they found the session useful and that they would recommend participation to others. People described the session as ‘enjoyable’, ‘helpful’, and ‘beneficial’, reporting that they appreciated the opportunity to express themselves and to meet others in the same position. Sharing stories and being encouraged to speak openly was reported to be inspiring and reassuring. Participants felt that others in their position would benefit from a similar session. These attitudes are described in the context of three subthemes, reported below.

#### ‘A positive response’

Participants emphasised the positivity they had felt during and after the session. People were quite surprised by how much they had enjoyed it and how optimistic it had made them feel.

“I left there feeling quite positive, and uplifted … ” (P2)

“I’d say positive, definitely positive … optimistic … yeah, just … kind of, all kind of good words … ” (P4)

#### ‘Comfort in connecting’

Asked to expand on what they had found helpful, participants referred to the comfort obtained from meeting others in the same position. A wish to ‘connect’ had been a particular motivation for attending for some.

“I don’t think it was just to sort of, well, you know, just come and see how it was and then go back home—there was deffo like, a massive reason to meet other people and to find out their stories and then, you know, to just compare them and to see, you know, how similar they were to my story.” (P3)

Participants strongly identified with the concept of ‘not being alone’, or that ‘others are the same as me’. The notion that others in their position were ‘normal’ and coping with their everyday lives helped to galvanise participants’ personal sense of identity as healthy individuals, and to modify their perceptions of being alone in their situation.

“ … there’s that same point of knowing that you’re not on your own, and there are other people there, you know I think, I think it’s still a good thing to do, even when you are struggling.” (P3)

#### ‘Reassurance and inspiration’

Participants spoke of improved confidence and optimism, with reference to their own personal lives, possibilities regarding group participation in the future and also progression of HD research.

“… it’s a chance just to involve people like yourself and listen to them, and you know, they’re hopeful, and you know, you read stuff and—it does it gives you a bit of a boost …” (P1)

“It was quite kind of, nice to actually [ … ] come out of it, sort of, feeling that actually, you know, yeah, I could take part in group sessions, and if,—if it ever came to that, or, you know if that was […] one of the things in the future, you know, there certainly wouldn’t be any fear going into a room full of people that I don’t know.” (P5)

Two participants expressed enhanced feelings of ambition and focus at work, voicing recognition of their current health status and motivation to capitalise on current opportunities.

“… I was just thinking about the future, and not letting anything drag me down, […] I wanted to be very ambitious and focused, rather than having to, you know, get […] depressed about things, it was about being positive”. (P4)

“I was kind of—I was almost like, quite inspired? To sort of afterwards, to almost try and even do, you know, like get more involved or do something a little bit more?” (P5)

One person described how the session had encouraged him to consider and compare the differences between his own position and that of his own father at his age, enabling him to appreciate his own potential.

“It really brought home that, you know, how healthy I am. Because you know, as a guy, with this gene, and I think my Dad, at this age, was—maybe I’m reaching the turning point because he died when he was, what, 48? So, you know … I’m glad I am where I am at the minute. You know, compared to being in his situation.” (P4)

Participants also spoke about their re-discovery of existing coping mechanisms, recognising their own agency in dealing with low mood.

“I think when I was a bit down I sort of had (partner) to pick me back up again, and then we’d sort of like, we’d go out for a meal or something, just to cheer myself up a bit, or I’d go and see my mum, and just sort of, let it all out on her.” (P3)

### Theme 3—recommendations for the future

All participants reported that they would consider attending a group session again. They suggested that the session could be applied in other circumstances, reflecting on the value for other family members, and people with other illnesses. Few suggestions were made for improvement, but they included re-consideration of the location and space, and beliefs around participant selection. Three subthemes were identified, discussed below.

#### ‘Positivity towards future sessions’

Although participants described feeling wary before the session, all indicated that they would be keen to attend future sessions.

“‘yeah, cause you know what it’s going to be like the second time don’t you. I know there’d be different faces but, probably. … […] if they were thinking of doing something different next time, it’d be interesting to find out”. (P6)

Several people reported that participation had helped to alleviate anxieties they had previously held about taking part in group activities. Participants viewed this increased confidence in the context of opportunities that they might benefit from in the future.

“I did feel like I’d be confident going to another one of them sessions”. (P2)

#### ‘Application to other circumstances/conditions’

Participants suggested that others could benefit from a similar type of group session. Whilst some considered how affected family members might benefit, others proposed that the sessions would be helpful for people with other conditions, such as cancer.

“Well, if there’s anyone else needing counselling, like, on similar subjects, but you found like a group of people, like you did with us, obviously, I don’t know, like say, with cancer patients or whatever, and you know, you just got ‘em speaking about their stories and their aspects of things and how they cope, you know with, when they’re having down days and stuff…” (P2)

“It would be good to see the outcome of doing something like that, just to see how people who are already going through the stages feel about it, and you know, how it compares to someone who has proven positive but hasn’t got the symptoms”. (P3)

#### ‘Suggested adaptations’

Six participants felt that the session could not have been improved or carried out in any other way. Two people considered ways in which their experience might have been improved by the selection of more similar co-participants. One reported feeling distanced from the group on the basis of age, whereas another would have welcomed the opportunity to have time exclusively with others who had been through the testing experience.

“So I would have just liked us—you know, all the people who had the … gene to just group together at one point perhaps, and just shared things, you know, and just you know, had their own little thing”. (P4)

The location of the session (within the Clinical Genetics Department) was discussed regarding its formality and possible prior clinical associations. One person expressed initial reservations, yet on reflection the informal nature of the session, within the context of a place that might have such a substantial meaning to people, was thought to potentially have a positive effect.

“I mean maybe thinking back now, it’s, it wasn’t as bad, because you know, by going into the, you know actually going into the clinic it can actually kind of reaffirms that it’s attached to that in some way”. (P5)

## Discussion

This study demonstrates an innovative approach to offering support following predictive testing for HD. It is the first study to apply narrative practices with people who have received a mutation positive HD predictive test result. In response to the literature illustrating fluctuating challenges of adjustment and family communication, we have created sessions that aim to nurture feelings of resilience and connectedness.

Participants emphasise the friendly, relaxed nature of the group and the importance of facilitated structure in providing a ‘safe’ space for self-expression. They valued the opportunity to listen and compare personal stories within a diverse group, an opportunity rarely afforded in everyday life. Although some people reported taking a relatively ‘open’ approach towards discussing HD, they recognised its ‘sensitive’ nature amongst family members and other individuals in the same position, highlighting this factor as preventative in expressing their own thoughts and feelings. A sense of ‘being alone’ was described following a positive test result; therefore, connecting with others and learning from them was a clear motivation for taking part. The narrative session was typically their first instance of communicating with others in a similar position. Participant accounts resonate with previous work describing changes in family communication and relationships (Tibben et al. 1993; Tibben et al. 1992) and reconstruction of roles and identities following predictive testing (Sobel and Cowan 2000; Tibben et al. 1997). Moreover, their positive reactions to the session echo the call for stimulation of open communication in post-test counselling (Decruyenaere et al. 2004), with approaches promoting open reflection and discussion of emotions, ideas and even painful experiences (Byng-Hall 1998; DudokdeWit et al. 1998).

Active use of narrative practices facilitated discussion of distressing life experiences without focusing on the ‘the problem’ (for example, HD in the family, or a mutation positive result). Rather the emphasis is on how a person resists the effects of that ‘problem’ and starts to change their relationship with it. Separation of the condition and person is a central tenet of narrative practices (White and Epston 1990), and encourages participants to question characteristics that might be seen as an inevitable part of the condition.

All respondents reported that they would recommend the session to others. There was positivity towards a repeat session, but nobody stated a need for this. This is important, as sessions were envisioned as standalone events with one-time attendance. Notably, many people who have had a predictive test for HD are of working age and have a range of responsibilities that prevent them from becoming engaged in a longer-term programme. Conspicuously, one of the only systematic programmes designed to help presymptomatic individuals cope with emotional stressors involves a structured set of training sessions, and reports low uptake and high drop-out rates (A'Campo et al. 2012), suggesting that long-term programmes may not be wholly viable for participants. Likewise, long-term support would incur significant cost and resources for service providers.

A further strength of the evaluation is that telephone interviews were carried out by an independent observer, and not by the session facilitators. This enabled participants to talk more openly about their experiences.

### Study limitations

This was an exploratory evaluation with a small, purposively selected sample. Limited demographic information was collected and it is unlikely that the participants were fully representative of this cohort. Whilst we recognise that we cannot generalise from this study, the aim of the evaluation was to ascertain the value and feasibility of employing these sessions within the genetic counselling context. These individuals described the session as useful, positive and motivating. This encourages us to extend the number of participants and types of groups offered, potentially allowing for a more representative sample.

The inclusion of partners in the group raises the question of whether differences were discernible between mutation positive individuals and partners. However, the limited data meant that meaningful comparison was not possible. This could also be an area for future study.

The quantitative measures, included due to their common use in primary care settings, did not indicate change. These self-report screening tools are designed to identify symptoms of depression and anxiety. It is conceivable that whilst participants do not identify with ‘pathological’ signs of mood disorder, they might still feel alone, or have difficulty coping with everyday uncertainties. Future work might benefit from including complimentary measures of resilience (Smith et al. 2008) and self-efficacy (Schwarzer and Jerusalem 1995) to capture more relevant quantitative outcomes.

This work responds to the post-test support objectives of the International Predictive Test Guidelines for HD (MacLeod et al. 2013). It outlines the importance of offering follow-up support, and proposes an exciting new approach for this in HD. The narrative techniques used can be valuable at any stage of the genetic counselling process, as they help people to recognise and rediscover their own sense of personal agency and coping mechanisms in the face of difficulties. Vitally, the project promotes multidisciplinary collaboration, demonstrating how genetic counsellors and practitioners from other specialties can work together to support people following predictive genetic testing in a structured way.

This study inspires us to consider new ways of talking about coping. Future research could usefully explore the use of narrative group sessions for individuals impacted by HD regardless of testing status. As two participants reported a preference for more similarity within participants’ characteristics (i.e. age, partners in a separate group), this could be considered in forthcoming recruitment, for example arranging groups specifically for younger people. It also raises the question of whether the approach could be adapted for other genetic conditions such as inherited cardiac disorders or cancer. A larger pilot study is ongoing to address these questions.
